# Supplementary material for: WordCommentsAnalyzer: A windows software tool for qualitative research
Source: F1000Res. 2018 Sep 4;7:536. Originally published 2018 May 3. [Version 2] doi: 10.12688/f1000research.14819.2 (PMC6137411; doi:10.12688/f1000research.14819.2)
Supplement: Supplementary file 3 [file f1000research-7-17614-s0002.tgz › b2a79496-4ada-47c4-9682-a99ce867836d.zip!/Eight Iranian Health Journals/Journal of Education and Community Health_4.docx]

© Journal of Education and Community Health. This work is licensed under CC BY-NC 4.0 (https://creativecommons.org/licenses/by-nc/4.0/).

Volume & Issue: دوره 4 - شماره 1

Title: بررسی عوامل مرتبط با رفتارهای پیشگیری کننده از سرطان پوست در دانش آموزان دبیرستانی شهر سنندج: کاربردی از الگوی پرسید

http://jech.umsha.ac.ir/article-1-309-fa.html

سابقه و هدف: در سال‌های اخیر، میزان شیوع سرطان پوست در ایران افزایش یافته است؛ به‌طوری که در استان کردستان شایع‌ترین سرطان می‌باشد. هدف از مطالعه حاضر، تعیین وضعیت رفتارهای پیشگیری‌کننده از سرطان پوست و عوامل مرتبط با آن در دانش‌آموزان دبیرستانی شهر سنندج با استفاده از الگوی پرسید (PRECEDE) بود. مواد و روش‌‌ها: مطالعه توصیفی- مقطعی حاضر در سال تحصیلی 1394-1393 روی 225 نفر از دانش‌آموزان (17-15 سال) مقاطع دوم و سوم دبیرستان در شهر سنندج که با روش نمونه‌گیری خوشه‌ای چندمرحله‌ای انتخاب شدند، صورت گرفت. این دانش‌آموزان پرسشنامه‌هایی شامل: سازه‌های آگاهی، نگرش، خودکارآمدی، حمایت اجتماعی، عوامل قادرکننده و رفتارهای پیشگیری‌کننده از سرطان پوست را به‌صورت خودایفا تکمیل کردند. در این مطالعه، داده‌ها با استفاده از SPSS 21 و آزمون‌های آنالیز واریانس یک‌طرفه،  Tمستقل، همبستگی پیرسون و رگرسیون خطی تحلیل گردید. یافته‌ها: براساس نتایج این مطالعه، همبستگی مثبت و معناداری بین رفتارهای پیشگیری‌کننده از سرطان پوست با متغیرهای نگرش (0.175=r)، خودکارآمدی (0.509=r)، حمایت اجتماعی (0.332=r) و عوامل قادرکننده (0.347=r) وجود داشت. همچنین میانگین انجام رفتار در دانش‌آموزان کمتر از متوسط بود. در تحلیل رگرسیون خطی، 29.9 درصد از واریانس رفتارهای پیشگیری‌کننده از سرطان پوست توسط متغیرهای مستقل پیش‌بینی گردید که در این میان، تنها متغیر پیش‌بینی‌کننده معنادار، خودکارآمدی (P<0.001 ,B=0.416) بود. نتیجه‌گیری: در مطالعه حاضر، خودکارآمدی درک‌شده برای انجام رفتارهای پیشگیرانه از سرطان پوست، یک عامل محوری بود. گفتنی است در مداخلات ارتقای رفتارهای پیشگیری‌کننده از سرطان پوست در میان دانش‌آموزان، تمرکز بر افزایش درک از خودکارآمدی انجام رفتار به‌عنوان یک راهبرد دارای اولویت پیشنهاد می‌شود.

Title: تعیین‌کننده‌های شناختی اجتماعی قصد آموزش به بیمار توسط پرستاران

http://jech.umsha.ac.ir/article-1-260-fa.html

سابقه و هدف: آموزش‌دادن به بیمار وظیفه‌ای مهم و یکی از استانداردهای کیفیت مراقبت پرستاری به‌شمار می‌آید و نقش بسزایی در بهبود بیماران و ارتقای سلامتی آنان دارد. مطالعه حاضر با هدف تعیین عوامل پیش‌بینی‌کننده قصد رفتار آموزش به بیمار در میان پرستاران با بهره‌گیری از نظریه رفتار برنامه‌ریزی‌شده صورت گرفت. مواد و روش‌‌ها: مطالعه توصیفی- مقطعی حاضر در میان 300 نفر از پرستاران بیمارستان‌های آموزشی- درمانی وابسته به دانشگاه علوم پزشکی و خدمات بهداشتی- درمانی شهر کرمانشاه انجام شد. در این مطالعه، شرکت‌کنندگان با روش نمونه‌گیری خوشه‌ای و به‌صورت تصادفی ساده با احتمال متناسب با حجم از بیمارستان‌های آموزشی- درمانی انتخاب شدند و اطلاعات با استفاده از پرسشنامه و به‌صورت خودگزارش‌دهی جمع‌آوری گردید. گفتنی است این پرسشنامه شامل دو بخش: جمعیت‌شناختی و سازه‌های نظریه رفتار برنامه‌ریزی‌شده در‌خصوص قصد آموزش به بیمار بود. داده‌های جمع‌آوری‌شده در نرم‌افزار SPSS 16 و با بهره‌گیری از آزمون‌های رگرسیون خطی، Chi-Square، آزمون T ، Anova و همبستگی پیرسون تحلیل شدند. یافته‌ها: در مطالعه حاضر، تعیین‌کننده‌های نگرش، هنجارهای ذهنی و کنترل رفتار درک‌شده درمجموع، 35 درصد از واریانس قصد رفتار آموزش به بیمار را در میان پرستاران پیش‌بینی کردند (0.35=R2). در این میان، نگرش پیش‌بینی‌کننده قوی‌تری بود (0.287=ß). همچنین قصد رفتار، همبستگی معناداری با هنجارهای ذهنی (P<0.001، r=0.470)، کنترل رفتار درک‌شده (r=0.384 ،P<0.001) و نگرش (P<0.001، r=0.508) داشت. نتیجه‌گیری: براساس نتایج مطالعه حاضر، به‌نظر می‌رسد تعیین‌کننده نگرش درخصوص آموزش به بیمار، تأثیر بیشتری بر قصد رفتار آموزشی پرستاران به بیماران داشته باشد؛ بنابراین پیشنهاد می‌شود در صورت نیاز به انجام اقدامات بیشتر، به این تعیین‌کننده توجه بیشتری شود.

Title: ارتباط بین رفتارهای خودمراقبتی و کیفیت زندگی در بیماران مبتلا به پرفشاری خون مراجعه کننده به مراکز جامع سلامت شهر همدان

http://jech.umsha.ac.ir/article-1-299-fa.html

سابقه و هدف: تبعیت‌نکردن از رفتارهای خودمراقبتی می‌تواند بر کیفیت زندگی بیماران مبتلا به پرفشاری خون تأثیر‌گذار باشد؛ بنابراین پژوهش حاضر با هدف بررسی رفتارهای خودمراقبتی در افراد مبتلا به پرفشاری خون و رابطه آن با کیفیت زندگی صورت گرفت. مواد و روش‌‌ها: پژوهش حاضر یک مطالعه توصیفی از نوع مقطعی بود که در سال 1396 روی 200 نفر از بیماران مبتلا به پرفشاری خون مراجعه‌کننده به مراکز جامع سلامت شهر همدان که به‌صورت نمونه‌گیری چندمرحله‌ای انتخاب شدند، صورت گرفت. ابزار گردآوری داده‌ها شامل: اطلاعات دموگرافیک، پرسشنامه‌های رفتارهای خودمراقبتی (ScBS: Self-Care Behaviors Scale) و استاندارد کیفیت زندگی (SF-36: Quality of Life) بود. داده‌های به‌دست‌آمده با استفاده از آزمون‌های T مستقل، آنالیز واریانس یک‌طرفه (ANOVA) و همبستگی پیرسون در نرم‌افزار SPSS 16 تجزیه و تحلیل گردید.  یافته‌ها: براساس یافته‌های به‌دست‌آمده، رفتارهای خودمراقبتی و ابعاد مختلف کیفیت زندگی به‌ترتیب با کسب 63.5 و 56.6 درصد حداکثر نمره قابل اکتساب در حد نسبتاً مطلوب و نامطلوب تقسیم‌بندی شدند و بین کیفیت زندگی با جنس، وضعیت تأهل و نمایه توده بدنی رابطه معناداری مشاهده شد (P<0.05). همچنین بین رفتارهای خودمراقبتی و سه بعد از کیفیت زندگی شامل: عملکرد جسمانی، عملکرد روانی و احساس درد و ناراحتی همبستگی مثبت و معناداری وجود داشت (P<0.05). گفتنی است بین نمرات کلی کیفیت زندگی و رفتارهای خودمراقبتی نیز همبستگی مثبت و معناداری مشاهده گردید (r=0.196، P<0.001). نتیجه‌گیری: با توجه به وجود ارتباط معنادار بین کیفیت زندگی مرتبط با سلامت و انجام رفتارهای خودمراقبتی، توصیه می‌شود برنامه‌های آموزشی برای بیماران مبتلا به پرفشاری خون به‌منظور افزایش تبعیت از رفتارهای خودمراقبتی طراحی و اجرا ‌گردد.

Title: اثربخشی امید درمانی گروهی برکیفیت زندگی و تاب آوری زنان معتاد

http://jech.umsha.ac.ir/article-1-256-fa.html

سابقه و هدف: پژوهش‌های انجام‌شده نشان می‌دهند که امید درمانی می‌تواند به‌عنوان مداخله روان‌شناسی مثبت‌گرا به کاهش مشکلات روان‌شناختی افراد کمک نماید؛ بنابراین مطالعه حاضر با هدف تعیین اثربخشی آموزش امیددرمانی گروهی بر کیفیت زندگی و تاب‌آوری زنان معتاد انجام شد. مواد و روش‌‌ها: پژوهش حاضر یک مطالعه نیمه‌تجربی از نوع پیش‌آزمون- پس‌آزمون همراه با گروه کنترل بود که با مشارکت 30 زن معتاد از کمپ شهرک وردآورد تهران انجام شد. در این مطالعه افراد مورد بررسی به‌طور تصادفی و براساس کسب نمره پایین در کیفیت زندگی و تاب‌آوری انتخاب و به دو گروه آزمون و کنترل تقسیم شدند. گفتنی است داده‌ها نیز با استفاده از پرسشنامه‌های‌ کیفیت زندگی فرم کوتاه (QOL: Quality of Life) و تاب‌آوری Connor و Davidson (CD-RISC: Connor-Davidson Resilience Scal) جمع‌آوری گردید. همچنین این داده‌ها با استفاده از روش آماری کوواریانس در نرم‌افزار 20 SPSS تجزیه و تحلیل شد. یافته‌ها: براساس نتایج این مطالعه، میانگین نمرات کیفیت زندگی (P<0.01، F=54.42) و تاب‌آوری (P<0.01، F=7.02) در مرحله پس‌آزمون در گروه آزمون افزایش معناداری را نشان داد؛ اما این تغییرات در گروه کنترل معنادار نبود. نتیجه‌گیری: براساس نتایج این مطالعه می‌توان گفت که آموزش امیددرمانی گروهی سبب افزایش امید و کیفیت زندگی در زنان معتاد و همچنین افزایش حرمت، خودپنداره مثبت و تقویت و تشویق برای بالفعل‌درآوردن توانایی‌های بالقوه مثبت در فرد می‌شود؛ بنابراین به درمانگران و متخصصان بهداشت روانی توصیه می‌شود که از این مداخله در کنار سایر درمان‌های دارویی و غیردارویی برای ارتقای سطح کیفیت زندگی و تاب‌آوری در برابر استرس زنان معتاد استفاده نمایند.

Title: پیشگویی کننده های اتخاذ رفتارهای پیشگیری کننده از مصرف سیگار در دانشجویان: کاربرد الگوی اعتقاد بهداشتی

http://jech.umsha.ac.ir/article-1-261-fa.html

سابقه و هدف: مصرف سیگار یکی از علل اصلی بروز بیماری‌های مختلف است. با توجه به روند افزایش مصرف سیگار در میان دانشجویان، مطالعه حاضر با استفاده از الگوی اعتقاد بهداشتی و با هدف تعیین پیشگویی‌کننده‌های اتخاذ رفتارهای پیشگیری‌کننده از مصرف سیگار در دانشجویان طراحی و اجرا شد. مواد و روش‌‌ها: پژوهش حاضر یک مطالعه توصیفی- تحلیلی بود که روی 340 نفر از دانشجویان ساکن در خوابگاه دانشگاه علوم پزشکی شهید بهشتی تهران که به روش نمونه‌گیری خوشه‌ای یک مرحله‌ای انتخاب شدند، صورت گرفت. در این پژوهش ابزار گردآوری اطلاعات، پرسشنامه محقق‌ساخته براساس الگوی اعتقاد بهداشتی بود. همچنین داده‌های به‌دست آمده با استفاده از آمارهای توصیفی، ضریب همبستگی پیرسون و آزمون رگرسیون چندگانه در نرم‌افزار 16 SPSS تجزیه و تحلیل شد. یافته‌ها: در این پژوهش، شرکت‌کنندگان 45.91 درصد از نمره اتخاذ رفتارهای پیشگیری‌کننده از مصرف سیگار را کسب کرده بودند. از بین متغیرهای مستقل این مطالعه، سازه‌های حساسیت درک‌شده و موانع درک‌شده کمترین نمره و سازه‌های منافع درک‌شده و خودکارآمدی بیشترین نمره را به خود اختصاص داده بودند. نتایج آزمون رگرسیون چندگانه نشان داد سازه‌های حساسیت درک‌شده و خودکارآمدی، پیش‌بینی‌کننده اتخاذ رفتار پیشگیری‌کننده از مصرف سیگار بودند. این متغیرها درمجموع، قادر به پیش‌بینی 24.6 درصد از تغییرات رفتار بودند. نتیجه‌گیری: براساس نتایج این مطالعه در طراحی مداخلات آموزشی باید بر متغیر‌های حساسیت درک‌شده و خودکارآمدی به‌عنوان مهم‌ترین پیشگویی‌کننده‌های اتخاذ رفتارهای پیشگیری‌کننده از مصرف سیگار در دانشجویان تأکید شود.

Title: بررسی مشارکت مالی خانوارهای شهری ایرانی در نظام سلامت در طی سال‌های 1383 تا 1395 : با تأکید بر طرح تحول نظام سلامت

http://jech.umsha.ac.ir/article-1-296-fa.html

سابقه و هدف: یکی از مهم‌ترین اهداف طرح تحول نظام سلامت، کاهش پرداخت از جیب خانوارها برای تأمین هزینه‌های سلامت می‌باشد. در این مطالعه مشارکت مالی خانوارهای شهری ایرانی در بخش سلامت قبل و بعد از طرح تحول مورد بررسی قرار گرفت. مواد و روش‌‌ها: پژوهش حاضر یک مطالعه توصیفی گذشته‌نگر می‌باشد. در این مطالعه پرداخت‌های بهداشت و درمان خانوارها با توجه به ویژگی‌های اقتصادی آن‌ها و با استفاده از داده‌های گزارش ﺑﻮدﺟﻪ ﺧﺎﻧﻮار در ﻣﻨﺎﻃﻖ ﺷﻬﺮی اﻳﺮان در سال‌های 1395-1383 مطالعه گردید. گفتنی است داده‌های به‌دست آمده با استفاده از آماره‌های توصیفی در نرم‌افزار 2016 Excel تجزیه و تحلیل شد. یافته‌ها: براساس یافته‌های به‌دست آمده مشاهده شد که هزینه‌های سلامت خانوارها از 11857892 ریال در سال 1391 با یک رشد قابل توجه به 16021227 ریال در سال 1392 و با رشدی ملایم‌تر به 19031440 ریال در سال 1393 رسیده است. همچنین گزارش شد که در دهک‌های درآمدی بالاتر، هزینه‌های سلامت نقش بزرگتری در هزینه‌های کل خانوار دارد؛ به‌طوری که بیش از 6.3 درصد هزینه‌های خانوار دهک دهم به سلامت اختصاص پیدا کرده است؛ درصورتی که این رقم در دهک اول کمتر از 4.8 درصد بوده است. نتیجه‌گیری: نتایج این مطالعه نشان داد که هزینه‌های سلامت در سال 1393 شیب کمتری نسبت به سال 1392 داشته است. این رشد در سال‌های 1394 و 1395 نیز کمتر از سال 1393 بوده است. اگرچه عوامل دیگر ازجمله: کاهش نرخ تورم نیز در کاهش نرخ رشد هزینه‌های سلامت خانوار مؤثر بوده؛ اما می‌توان طرح تحول سلامت را نیز در این مورد تأثیرگذار دانست.

Title: فاکتورهای مرتبط با انجام آزمایش پاپ‌اسمیر در بین زنان مراجعه‌کننده به مراکز بهداشتی-درمانی شهر فسا: کاربرد نظریه رفتار برنامه‌ریزی‌شده

http://jech.umsha.ac.ir/article-1-285-fa.html

سابقه و هدف: سرطان سرویکس دومین سرطان شایع در بین زنان می‌باشد. این سرطان به‌دلیل دوره طولانی قبل از تهاجم، برنامه غربالگری مناسب و درمان مؤثر ضایعات اولیه، یک سرطان قابل پیشگیری می‌باشد. مطالعه حاضر با هدف بررسی فاکتورهای مرتبط با انجام آزمایش پاپ اسمیر براساس نظریه رفتار برنامه‌ریزی‌شده روی زنان شهر فسا انجام شد. مواد و روش‌‌ها: پژوهش حاضر یک مطالعه توصیفی- تحلیلی و از نوع مقطعی بود که در سال 1395 روی 700 نفر از زنان متأهل که به‌صورت تصادفی ساده از بین مراجعان به مراکز بهداشتی- درمانی شهر فسا انتخاب شده بودند، انجام شد. ابزار گردآوری داده‌ها، شامل اطلاعات جمعیت‌شناختی، پرسشنامه آگاهی و سازه‌های نظریه رفتار برنامه‌ریزی‌شده (Theory of Planned Behavior) بود. همچنین داده‌ها با استفاده از آزمون‌های ضریب همبستگی پیرسون و آنالیز رگرسیون لجستیک در نرم‌افزار 22 SPSS تحلیل شد. یافته‌ها: در این مطالعه، 45.7 درصد از زنان سابقه انجام آزمایش پاپ اسمیر را گزارش نمودند و 20.7 درصد نیز به‌طور مرتب آزمایش پاپ اسمیر را انجام می‌دادند. طبق یافته‌های به‌دست آمده، سازه‌های آگاهی، نگرش، کنترل رفتاری درک‌شده و هنجارهای انتزاعی پیش‌بینی‌کننده قصد و رفتار غربالگری آزمایش پاپ اسمیر در زنان بودند (0.05>P). این سازه‌ها، 57.4 درصد از واریانس قصد و 31.6 درصد از واریانس رفتار را پیشگویی می‌کردند. نتیجه‌گیری: با توجه به نتایج مطالعه حاضر، می‌توان با برنامه‌ریزی مناسب در خصوص افزایش آگاهی، بهبود نگرش و هنجارهای انتزاعی و افزایش کنترل رفتاری درک‌شده، میزان انجام رفتارهای غربالگری را در بین زنان ارتقا داد که این خود در پیشگیری و کنترل سرطان دهانه رحم نقش بسزایی دارد.

Title: مقایسه سازه‌های الگوی اعتقاد بهداشتی بر حسب انتخاب روش زایمان در زنان نخست زای شهر بندرعباس

http://jech.umsha.ac.ir/article-1-294-fa.html

سابقه و هدف: عمل سزارین گسترده‌ترین عمل جراحی برای زایمان در جهان می‌باشد و میزان مرگ و میر ناشی از آن، 7 برابر زایمان طبیعی است. مطالعه حاضر با هدف مقایسه سازه‌های الگوی اعتقاد بهداشتی بر حسب انتخاب روش زایمان در زنان نخست‌زای شهر بندرعباس انجام شد. مواد و روش‌‌ها: پژوهش حاضر یک مطالعه توصیفی- تحلیلی و از نوع مقطعی بود که روی 210 مادر باردار نخست‌زای مراجعه‌کننده به مراکز بهداشتی- درمانی شهر بندرعباس در سال 1395 انجام شد. روش نمونه‌گیری ترکیبی از روش‌های خوشه‌ای و تصادفی ساده بود. ابزار جمع‌‌آوری داده‌ها، پرسشنامه سه قسمتی شامل اطلاعات جمعیت‌شناختی، آگاهی و سازه‌های الگوی اعتقاد بهداشتی بود. داده‌های به‌دست آمده با استفاده از آماره‌های توصیفی و آزمون‌های Chi square و آنالیز واریانس یک‌طرفه در نرم‌افزار 18  SPSS تحلیل شد. یافته‌ها: براساس یافته‌های به‌دست آمده، میانگین سنی مادران باردار شرکت‌کننده در این پژوهش، 4.96±24.56 سال بود. 63.8 درصد از مادران زایمان طبیعی و 36.2 درصد عمل سزارین انجام داده بودند. همچنین میانگین نمره آگاهی مادران از مزایا و معایب روش‌های زایمانی، 63/6 درصد برآورد گردید. اختلاف آماری معنا‌داری نیز در تمامی سازه‌های الگوی اعتقاد بهداشتی بین روش‌های زایمان انتخابی مادران مشاهده شد (P<0.05). نتیجه‌گیری: با توجه به نتایج حاصل از این مطالعه، برگزاری مداوم کلاس‌های آموزشی در زمینه عوارض و خطرات سزارین توسط مراکز بهداشتی در کنار مراقبت‌های دوران بارداری، شناسایی موانعی که برای انتخاب زایمان طبیعی بر سر راه مادران قرار گرفته و اقدام در برطرف‌کردن آن‌ها با هدف اعتماد هرچه بیشتر مادران برای انجام زایمان به روش طبیعی می‌تواند در کاهش عمل‌های سزارین‌ غیرضروری مؤثر واقع شود.
